# Welcome to *Biotechnology for Biofuels*

**DOI:** 10.1186/1754-6834-1-1

**Published:** 2008-04-15

**Authors:** Bärbel Hahn-Hägerdal, Michael E Himmel, Chris Somerville, Charles Wyman

**Affiliations:** 1 Department of Applied Microbiology, LTH/Lund University, PO Box 124, S-221 00, Lund, Sweden; 2 Chemical and Biosciences Center, National Renewable Energy Laboratory, Cole Boulevard, Golden, CO 80401-3393, USA; 3 Energy Biosciences Institute, University of California Berkeley, 130 Calvin Laboratory, MC 5230, Berkeley CA 94720-5230, USA; 4 Center for Environmental Research and Technology (CE-CERT), Bourns College of Engineering University of California, Columbia Avenue, Riverside, CA 92507, USA

## Introduction

We are pleased to announce a new open-access journal, *Biotechnology for Biofuels *[1], published online by BioMed Central. *Biotechnology for Biofuels *will emphasize the research and application of biotechnology and synergistic operations to improve plant and biological conversion systems for the production of fuels from lignocellulosic biomass, and any related economic, environmental and policy issues.

The need for this journal is evident: the recent explosion in research on the production and subsequent use of biofuels has huge implications for science and future policy directions, yet *Biotechnology for Biofuels *is the first open-access journal featuring research dedicated to this exciting and expanding field, thereby filling a vacant niche. We are convinced that a communication will facilitate scientific progress in this extremely important area, and will also help to promote informed public debate. *Biotechnology for Biofuels *will ensure public availability of high-caliber peer-reviewed research, reviews and commentaries on all aspects of biofuels research and any related political, economic, and environmental issues.

The benefits of publishing in an open access journal are manifold: open access enables free and universal access to articles online, at no cost to the reader, allowing research to be disseminated by as wide an audience as possible. Submitted manuscripts undergo rapid peer review by internationally renowned experts, drawn in part from our Editorial Board [2]. Articles are published immediately upon acceptance and, soon after, listed in PubMed [3]; the communication of research is therefore not postponed until the collation of an 'issue'.

The interdisciplinary nature of biofuels research makes the benefits of open access particularly attractive, as it ensures that biologists, chemists, engineers, genomicists, and biotechnologists (to name just some of those involved) all have shared access to the latest biofuels research in each of these areas.

In this special Editorial, which marks the launch of *Biotechnology for Biofuels*, the progress and future challenges facing the biofuels field are discussed.

## Industrial outlook for biomass conversion to fuels and chemicals

Industrial interest in commercializing biomass conversion to fuels and chemicals has grown so fast of late that it would be futile to list all of the players in the field without the risk of some important omissions. Furthermore, the diversity of biological technologies being pursued is broad, with targets including faster growing crops, crops more amenable to conversion, advanced pretreatments, better enzymes, as well as improved organisms for production of ethanol, butanol, other alcohols, alkanes, and diesel fuel substitutes. There are a number of important reasons behind this surge in interest. First, despite historically low funding for research and development, significant advances have been made in some key areas through programs led by agencies around the world. Particularly noteworthy progress has been made in improving technologies for biological conversion of cellulosic biomass through better pretreatments, enzymes, and fermentative organisms. Consequently, the economics of biological conversion processes has improved. Second, recent dramatic oil price increases have enhanced the competitiveness of alternative fuels. Third, these high oil prices have finally prompted governments to adapt policies that encourage commercialization. Fourth, mounting concerns about global climate change and the need for carbon-neutral fuels have narrowed the options for the large-scale, low-cost production of organic liquid fuels and chemicals to be used as biomass. Finally, biological processes are of particular interest because of the opportunities to apply the powerful tools of modern biotechnology to dramatically reduce costs and improve the efficiencies of both producing biomass and converting it into organic fuels and chemicals.

Although commercial momentum is building, it is vital to keep in mind that significant challenges still face the emergence of this industry. Capital costs for biofuels facilities are high, as is typical of most sustainable technologies, and will be particularly high for first applications. In addition, because the technology is not proven at a large scale, investors expect very high rates of return for initial projects, that is, a high cost for borrowing the capital needed. Concerns about risk are further magnified by the tight margins for making fuels and other commodity products. Thus, building this industry faces classic chicken-and-egg problems. On top of this, the press periodically sensationalizes possible consequences of biofuels use, spooking investors and deterring interest. Consequently, strategies are needed to reduce the risk of the first few applications, including the use of low-cost residues as feedstocks, integration into existing facilities, access to low-cost debt, sale of valuable co-products, employment of a commercialization team with a successful track record, and the use of existing technology where possible. However, these measures are likely to prove inadequate, and aggressive government policies are needed to promote the construction of the first few facilities in a way that meets the needs of both investors and society. In addition, improving our understanding of the fundamental principles governing the production and conversion of lignocellulosic biomass and rapid dissemination of that knowledge through journals such as *Biotechnology for Biofuels *are invaluable in reducing the risk of the first applications and providing a foundation for technical advances that significantly lower costs.

## Outlook for bioenergy crop improvements

Relatively little effort has been invested until now in addressing the key questions concerning the source of feedstocks for lignocellulosic biofuels production. One class of questions concerns the development of methods for collecting and transporting corn stover or other crop residues and investigations of how much residue can be removed from fields without reducing sustainability. A second, and much more complex class of questions concerns the identification and characterization of dedicated energy crops. What species are promising in various climatic zones and soil types? What are the best agronomic practices? How can they be propagated, harvested, stored, and transported? How much genetic variability is present for yield, composition, and response to biotic and abiotic stresses? Are there issues concerning sustainability? What are the effects of long-term cultivation and biomass removal on soil quality, runoff, erosion, and greenhouse gas emissions? Perhaps above all, what are the probable delivered costs for various biomass sources? Much of the long-term planning that is necessary in order to develop conversion facilities will depend on having realistic projections about the long-term availability of biomass at predictable unit costs. Finally, there is also broad interest in the idea that dedicated biomass crops may ultimately be engineered for improved conversion efficiency. The development of plants with modified lignin composition has been, and will continue to be, an important area of research with important implications for process development. Understanding the role of polysaccharide acetylation and the degree to which it can be genetically modified may also prove to be important. Indeed, essentially any basic information that allows a deeper understanding of the structure and function of plant cell walls and how walls can be modified by rational methods will be of long-term importance to the development of optimized feedstocks.

## Importance of the bioenergy field

To improve domestic energy security, begin to address greenhouse gas emission concerns, and reduce America's future demand for oil, aggressive goals for THE deployment of biofuels have been set in the United States by the Administration and Congress. President Bush has stated a near term goal of '20 in 10' (20% reduction in domestic gasoline use in the next 10 years, of which 75% of the goal will be met by alternative fuels) and a long-term goal of '30 × 30' (supplying 30% of 2030 light vehicle transportation fuel needs with biofuels). Similarly, Congress recently enacted the Energy Independence and Security Act (EISA) in December 2007. This act mandates 36 billion gallons of renewable fuels production per year by 2022 of which 21 billion gallons must be advanced biofuels.

Similar policies have been developed worldwide. For example, the EU Biofuels Directive of 2003 focused on the use of renewable fuels for transport, and set a target for Member States of a 5.75% market share for biofuels in transportation fuel by 2010, rising to 10% by 2020. In order to achieve these goals, the EU Strategy for Biofuels has focused on seven policy areas:

1. Stimulating demand for biofuels.

2. Capturing environmental benefits.

3. Developing the production and distribution of biofuels.

4. Expanding feedstock supplies.

5. Enhancing trade opportunities.

6. Supporting developing countries.

7. Supporting research and development.

In China, which has seen a rapid increase in demand for fuel, the National Development and Reform Commission (NDRC) oversees production and consumption of biofuels. The Chinese Government has set clear goals, whereby biofuels should constitute 15% of transportation fuels by 2020. In 2005, 1 million tons (approximately 340 million gallons) of bio-ethanol was produced from food grains as feedstocks; by 2020, China aims to utilize 10 million tons (3.4 billion gallons) of bio-ethanol, and a further 2 million tons (680 million gallons) of biodiesel from non-food feedstock, in order to meet its transportation needs.

Biomass is the only domestic and renewable primary energy resource that can provide liquid transportation fuels on a long-term sustainable basis. A long-standing question has been 'What is the production potential of lignocellulosic ethanol and can it have a significant impact on imported oil displacement and long-term energy security?'. In response to this question, an in-depth study 'Biomass as feedstock for a bioenergy and bioproduct industry: the technical feasibility of a billion-ton annual supply' [4] (the so-called 'billion ton study') was performed. This study estimated that the US has the potential to produce up to 1.3 billion tons of biomass annually on a sustainable basis without having an impact on food, feed, or fiber uses. To put the ethanol production potential from this amount of biomass plus grain-based ethanol in perspective, almost 60% of 2004 motor gasoline demands on an equivalent energy adjusted basis could be met with ethanol from grain and biomass. A key aspect of realizing the potential of biofuels is to address the sustainability issue, and all future development of biofuels needs to have sustainable development as a central theme.

## The need to have low-cost sugars

Basically, biochemical conversion of lignocellulosic biomass to ethanol can be described as the fermentation of sugars liberated from the feedstock. Challenges are to most efficiently convert biomass to sugars (saccharify) and then ferment these impure sugars to ethanol with a robust microorganism. Evolutionary processes, however, have effectively conspired to protect the sources of fermentable sugars in plants, structural polysaccharides of the cell wall, from deconstruction. Naturally occurring, fermentative microbial strains are also far from optimized for efficient and low-cost production of ethanol and other liquid fuels. Even with these challenges, this conversion process shows great promise for the cost-effective production of ethanol at high yields with minimal environmental impact, with the keys being low-cost pre-treatment and hydrolysis technologies to realize low-cost sugars

Market analysis has shown that critical to achieving the EISA and '30 × 30' goals is achieving near-term cost competitiveness of lignocellulosic ethanol defined as achieving a US$1.33/gallon production cost of lignocellulosic ethanol by 2012 [5]. Achieving this production cost target, which is benchmarked against projections for crude oil prices, will enable the start of a viable industry converting lignocellulosic biomass to ethanol. However, the market goals set in EISA of 36 billion gallons of renewable fuels production per year by 2022 and the long-term goal of displacing 30% of 2004 gasoline demands with biofuels by 2030 will require additional advancements in science and technology with continued reduction in feedstock supply system and processing costs. Future research and development efforts will be focused on complementary approaches to achieve the volumetric liquid fuels goal.

It is now clear that new translational science concepts will be adapted to make these advancements a reality. This approach is familiar to the biomedical industry and describes the integration of basic research (fundamental biological science) with the industrial application of technology (bioengineering). Regardless of the technical topic, fundamental science must be guided by applied objectives to ensure success. To meet the 2030 technical targets, a significant base of new fundamental science must be completed, and *Biotechnology for Biofuels *will aim to provide a publication platform that is consistent with this need.

## Fermentation challenges

The bioconversion of renewable resources to fuels and chemicals poses a number of challenges for the fermentation industry and for the development of novel industrial catalysts, enzymes, and microorganisms. The fermentation substrate is heterogeneous including not only carbohydrates but also lipids and peptides, it may be lacking essential nutrients, and may have suboptimal pH and temperature as well as high osmolality conditions which are commonly referred to as stress-inducing. The production scale of fuels and chemicals from renewable resources requires both simplified and sustainable bioconversion technology, which needs to be integrated with cost-effective and energy-saving downstream processing.

Microbial fermentation as a research field is presently being revolutionized conceptually, because of the explosion of available microbial genome sequences and the many technological developments that make it possible to translate this information into traits, which add value to the fermentation industry. The '-omics' technologies, including global transcription, protein, metabolite, flux analyses, and so on, as well as mathematical modeling of biological reactions, have been successfully implemented in applications related to human health. These novel technologies are now available to be integrated with traditional biocatalyst development such as breeding, mutagenesis, and adaptation. Also, newly developed catalysts, enzymes, and cells have to be benchmarked in an industrial context to identify novel targets for catalyst improvement. Of fundamental importance are technologies that allow *in situ *and *in vivo *monitoring of the performance of catalysts, enzymes, and cells in the industrial environment.

Overall, the challenges surrounding biofuels are huge, but given the rapid advances in the field we are convinced that we will see exciting changes. We truly believe that *Biotechnology for Biofuels *will facilitate this progress, and serve the biofuels research community. We look forward to the success of this new journal, and urge you to submit your next exciting research article to *Biotechnology for Biofuels*.
